# Workplace neighbourhood built-environment attributes and sitting at work and for transport among Japanese desk-based workers

**DOI:** 10.1038/s41598-021-03071-8

**Published:** 2022-01-07

**Authors:** Chien-Yu Lin, Mohammad Javad Koohsari, Yung Liao, Kaori Ishii, Ai Shibata, Tomoki Nakaya, Gavin R. McCormack, Nyssa Hadgraft, Takemi Sugiyama, Neville Owen, Koichiro Oka

**Affiliations:** 1 Graduate School of Sport Sciences, Waseda University, 2-579-15 Mikajima, Tokorozawa, Saitama 359-1192 Japan; 2 Faculty of Sport Sciences, Waseda University, Tokorozawa, Japan; 3 Melbourne School of Population and Global Health, The University of Melbourne, Melbourne, Australia; 4 Department of Health Promotion and Health Education, National Taiwan Normal University, Taipei, Taiwan; 5 Faculty of Health and Sport Sciences, University of Tsukuba, Tsukuba, Japan; 6 Graduate School of Environmental Studies, Tohoku University, Sendai, Japan; 7 Department of Community Health Sciences, Cumming School of Medicine, University of Calgary, Calgary, Canada; 8 Centre for Urban Transitions, Swinburne University of Technology, Melbourne, Australia; 9 Behavioural Epidemiology Laboratory, Baker Heart & Diabetes Institute, Melbourne, Australia

**Keywords:** Health occupations, Environmental social sciences

## Abstract

Workplace settings—both internal and external—can influence how workers are physically active or sedentary. Although research has identified some indoor environmental attributes associated with sitting at work, few studies have examined associations of workplace neighbourhood built-environment attributes with workplace sitting time. We examined the cross-sectional associations of perceived and objective workplace neighbourhood built-environment attributes with sitting time at work and for transport among desk-based workers in Japan. Data were collected from a nationwide online survey. The Abbreviated Neighborhood Environment Walkability Scale (n = 2137) and Walk Score® (for a subsample of participants; n = 1163) were used to assess perceived and objective built-environment attributes of workplace neighbourhoods. Self-reported daily average sitting time at work, in cars and in public transport was measured using a Japanese validated questionnaire. Linear regression models estimated the associations of workplace neighbourhood built-environment attributes with sitting time. All perceived workplace neighbourhood built-environment attributes were positively correlated with Walk Score®. However, statistically significant associations with Walk Score® were found for sitting for transport but not for sitting at work. Workers who perceived their workplace neighbourhoods to be more walkable reported a longer time sitting at work and in public transport but a shorter sitting time in cars. Our findings suggest that walkable workplace neighbourhoods may discourage longer car use but have workplaces where workers spend a long time sitting at work. The latter finding further suggests that there may be missed opportunities for desk-based workers to reduce sitting time. Future workplace interventions to reduce sitting time may be developed, taking advantage of the opportunities to take time away from work in workplace neighbourhoods.

## Introduction

Socio-ecological models suggest that urban design factors can play an important role in influencing physically active and sedentary behaviours^1,2^. Building on a growing body of evidence on urban design attributes related to physical activity, a recent science advisory statement from the American Heart Association recognised the role of activity-supportive neighbourhood built environment design in promoting active living and improving population health^3^. Such urban design strategies may have relevance for desk-based workplaces, where many workers spend most of their work hours sitting^4,5^. In addition to the indoor environment of such workplaces, in which some characteristics are known to be related to workers’ sitting time^6,7^, the external environment surrounding the workplace may also be relevant in this context. Given that occupational sitting tends to be accumulated in long bouts^5,8^, workplace neighbourhoods with opportunities for walking or to otherwise being away from the office environment may be conducive to lower amounts of sitting at work.

Two recent reviews have synthesised findings on the associations of workplace neighbourhood built-environment attributes with workers’ sedentary and active behaviours^9,10^. Zhu et al.^9^ found inconclusive evidence for associations of physical activity with most workplace neighbourhood attributes such as population density and access to destinations. Lin et al.^10^ found that longer distances from the workplace to home and better access to car parking around the workplace were associated with longer time spent driving to/from work. However, the majority of previous studies of workplace neighbourhood built-environment attributes examined physical activity but not sedentary behaviour. Furthermore, the studies on sedentary behaviour and workplace neighbourhoods examined sitting in cars rather than sitting at work. Previous reviews have shown that the relevant environmental correlates may differ between different domains of sedentary behaviour^11^. Identifying correlates of sedentary behaviours at work and for transport that add to the total volume of daily sedentary time can assist in developing health promotion strategies relevant to desk-based workers.

Few studies have investigated the associations between the workplace neighbourhood built environment and physical activity and sedentary behaviour in the Asian context. In their review, Lin et al.^10^ found that only three out of 55 studies were conducted in Asian countries, of which all three studies investigated physical activity. Evidence from Asian countries is needed, as workplace cultures and contexts are likely to differ between Asian and Western countries. One such difference is hours spent at work: the average work hours per week in workers, both full-time and part-time employed, from Asian countries such as China (46 h), South Korea (40 h), and Japan (38 h) are longer than the hours in Western countries such as the USA (37 h) and the UK (36 h)^12^. Another difference is the built environment where the workplace is situated. Compared with cities in Western countries, built environment patterns (e.g., street network, land use, and access to public transport) are likely to be different, primarily due to the higher population densities in Asian countries^13^. Such differences between Western and non-Western cities warrant investigation of workplace neighbourhood environments and transport-related sitting in an Asian context.

This study examined the associations of perceived and objective measures of workplace neighbourhood built-environment attributes with workplace and transport-related sedentary behaviours among desk-based workers in Japan.

## Methods

This study follows the STrengthening the Reporting of OBservational studies in Epidemiology (STROBE) guidelines for reporting observational studies.

### Participants

Data from an online survey conducted through a Japanese internet research service company (MyVoice Communication, Inc. Tokyo, Japan) in February 2019 was used for the study. This company retains some one million individuals across Japan voluntarily registered as panel members with detailed socio-demographic data. Potential participants aged 20–59 years with full-time jobs (n = 45,659) were randomly selected from the database and received an invitation email about this study via their company’s system. These potential participants were equally stratified by gender and age groups (20–29, 30–39, 40–49, and 50–59 years) to minimise the possibility of selection bias due to being overrepresented in specific demographic subgroups. They received an email invitation with a specific link to access an online questionnaire. A total of 3200 workers signed an online informed consent form and completed the questionnaire (response rate = 7.0%). Each participant received reward points valued at 1.5 USD as an incentive after they completed the survey. The analysis was limited to the participants who reported desk-based employment (n = 2265). All methods used in this study were in accordance with the relevant ethical guidelines. The Institutional Ethics Committee of Waseda University (2020–135) approved this study.

## Measures

### Outcome variables

#### Sedentary behaviours

A validated Japanese questionnaire^14^, which seeks to assess sedentary time in six specific behaviours across three domains (related to work, transport, and leisure) separately for workdays and non-workdays, was used (see Supplementary Table 1 for the full questionnaire). Participants were asked to report their daily average sedentary time for each behaviour over the previous week. We used three specific sedentary behaviours on workdays as the outcome measures: sitting time at work; sitting time in cars; and sitting time in public transport. These behaviours were considered to occur in or near participants’ workplace neighbourhoods, as workers carried them out on workdays. This questionnaire has shown moderate to high test–retest reliability (intraclass correlation coefficient [ICC] = 0.83) for the work domain with a 1-week recall period^14^. The criterion validity of all-domain sedentary time for workdays (rho = 0.57, p < 0.001) and the whole week (rho = 0.49, p < 0.001), comparing the questionnaire with accelerometer, is moderate^14^.

### Exposure variables

#### Perceived workplace neighbourhood built environment

The Abbreviated Neighborhood Environment Walkability Scale Japanese version (ANEWS-J) was used to measure environmental perceptions in the workplace neighbourhood. Workplace neighbourhood was defined as within a 10- to 15-min walk from the workplace. A total of six subscales were assessed: land use mix diversity (16 items), land use mix access (6 items), street connectivity (3 items), availability and quality of walking/cycling infrastructures (4 items), aesthetics (4 items), and crime safety (5 items). The Cronbach’s α, an indicator of internal consistency, for land use mix diversity, land use mix access, street connectivity, availability and quality of walking/cycling infrastructures, aesthetics, and crime safety were 0.91, 0.65, 0.64, 0.72, 0.73, and 0.56, respectively. We did not include the subscales of residential density, which was not applicable to the study, and traffic safety due to low internal consistency (α = 0.26)^15^. The details of the modified ANEWS-J used in this study were provided in Supplementary Table 2. All subscale items were rated on a four-point scale, except for those to assess land use mix diversity (six-point scale). Scoring the subscales followed the procedures of ANEWS-J published online (http://www.tmu-ph.ac/pdf/ANEWS_Jpn_ver3.pdf). Higher scores indicate greater walkability. ANEWS-J has been found to have acceptable test–retest reliability (ICCs = 0.76–0.96) for residential neighbourhoods^16^. We examined the test–retest reliability of ANEWS-J for workplace neighbourhood in a subsample of participants (n = 200). Participants reported their perceptions of their workplace neighbourhood environment twice within two weeks. The test–retest reliability of ANEWS-J was moderate to high for all subscales (ICC = 0.57–0.87) (Supplementary Table 3).

#### Objectively measured workplace neighbourhood walkability

The level of walkability in workplace neighbourhoods was estimated using Walk Score®. It is a measure of access to local destinations, using a distance-decay function to destinations such as grocery stores, restaurants, banks, parks, and schools, with adjustment by two street connectivity metrics: intersection density and block length^17^. Walk Score® can be assigned to locations (e.g., postcodes or addresses) and is normalised between 0 and 100. A higher Walk Score® indicates that there are more destinations within walking distance. Walk Score® uses open-source data such as Google, Education.com, and Open Street Map as the source data to identify relevant destinations^17^. Walk Score® has been confirmed as a valid measure to assess neighbourhood walkability in Japan^18^. Around 60% of the participants provided their seven-digit workplace postcodes (n = 1360), with 777 unable to provide their complete workplace postcodes. Each workplace postcode was manually entered into the Walk Score® website (www.walkscore.com) to obtain the score in July–August 2020. Walk Score® was available for 1163 participants. The website did not generate Walk Score® for 197 participants who provided a workplace postcode due to the limited data for spatial details from Japan. Since Walk Score® was negatively skewed (median score = 82, 25th percentile = 63, 75th percentile = 94), we used Walk Score® as a categorical measure. We classified participants into three groups according to Walk Score®: car-dependent (0–69); somewhat walkable (70–89); and very walkable (90–100).

### Covariates

Individual-level covariates included gender, age group (20–29, 30–39, 40–49, or 50–59 years), marital status (not married or married), educational level (tertiary education or below tertiary education), individual annual income (< 4,000,000 or ≥ 4,000,000 yen), physical activity duration, and work hours per week. We used the Global Physical Activity Questionnaire (GPAQ)^19^ to assess the amount of physical activity in three domains (work, transport, and leisure). The GPAQ data were checked for valid responses following the standardised procedures provided by the World Health Organization^20^. The total amount of physical activity for these domains was used as a covariate. Four more participants were excluded due to missing data on total physical activity. Information on the possession of a driving licence (yes/no) was also collected for transport-related sedentary behaviours. The work hours were assessed using the question “How many hours have you worked in the last 7 days?” The workplace-level covariate was workplace size, which was measured by the self-reported number of workers in the participant’s workplace (< 29, 30–99, ≥ 100 employees, or missing).

### Statistical analysis

Differences in characteristics across subsample categories were examined using Pearson’s Chi-square tests for categorical variables and independent t-tests for continuous variables. Spearman’s correlation was used to examine the correlations between perceived workplace neighbourhood built-environment attributes and Walk Score®, as the latter was skewed.

We used linear regression models to investigate the associations of workplace neighbourhood attributes with sedentary behaviour at work. The unstandardised regression coefficients (β) and 95% confidence intervals (CIs), corresponding to one standard deviation (SD) increment of perceived environmental attributes, were estimated for the associations. We also calculated β and 95% CI for the Walk Score® category, using the mid category (somewhat walkable) as the reference. Each workplace neighbourhood attribute was examined individually in the models. All regression analyses were conducted using Stata 15 (Stata Corp, College Station, Texas, USA), and the level of significance was set at p < 0.05.

## Results

**Figure 1:**
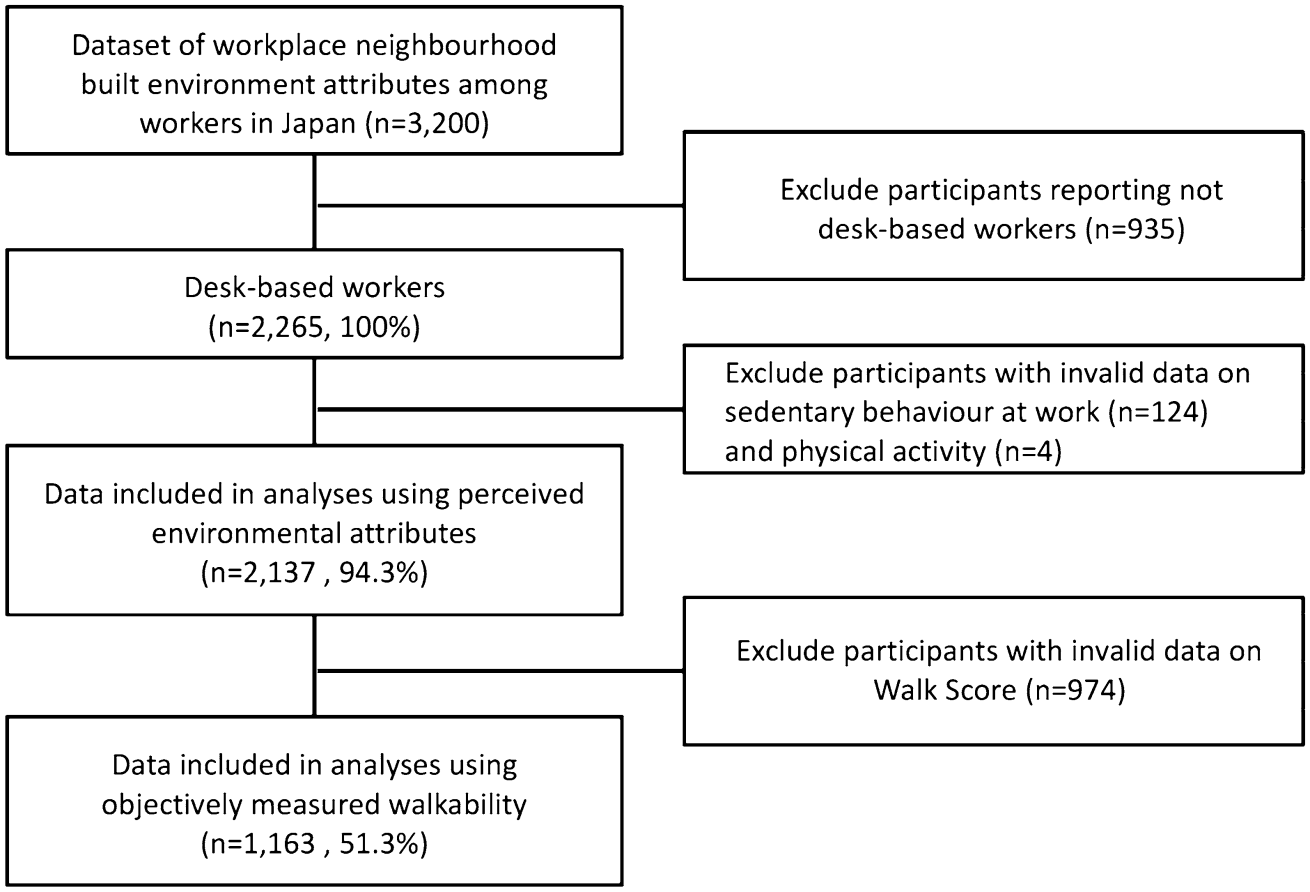
Flow chart of the analysed participants.

Valid responses on the workplace neighbourhood built-environment attributes and sedentary time at work were categorised into the overall sample (2137 desk-based workers reporting environmental perceptions) and Walk Score® subsample (1163 providing workplace postcodes that generated valid Walk Score®) (see Fig. 1). The majority of the participants (80%) were company employees. Participants’ characteristics for the overall sample and Walk Score® subsample were shown in Table 1. Participants spent on average about 6 h and 20 min per day sitting at work. Nearly 70% of the workplaces reported by those providing valid workplace postcodes were located in walkable neighbourhoods. There were no differences in the socio-demographic characteristics and environmental perceptions between the overall sample and the Walk Score® subsample. However, the subsample for which Walk Score® was available had a higher proportion of participants working in smaller workplaces than the overall sample. The comparison in characteristics between those who reported valid Walk Score® (n = 1163) and those who reported missing/invalid Walk Score® (n = 974) was also shown in Supplementary Table 4. As shown in Table 2, all perceived workplace neighbourhood built-environment attributes were positively correlated with Walk Score®—the strongest correlation was found for land use mix access (rho = 0.50), and the weakest correlation was found for aesthetics (rho = 0.12).

**Table 1:** Characteristics of participants and workplace neighbourhood environments.

Characteristics	Overall sample (n = 2137)	Walk Score® subsample (n = 1163)	p^a^
Gender, % men	48.0	50.1	ns
**Age group, %**			ns
20–29 years	23.6	20.9	
30–39 years	24.9	23.9	
40–49 years	25.7	27.5	
50–59 years	25.7	27.7	
Married, %	44.5	47.5	ns
Have tertiary education, %	86.0	86.4	ns
Annual income ≥ 4,000,000 yen, %	48.5	51.0	ns
Have driving licence, %	89.7	90.2	ns
Work hours per week, mean (SD)	44.7 (13.3)	45.0 (13.8)	ns
**Workplace size, %**			0.004
Small (≤ 29 workers)	23.9	29.7	
Medium (30–99 workers)	14.5	13.8	
Large (≥ 100 workers)	58.4	53.8	
Missing	3.2	2.8	
Total physical activity (h/day), mean (SD)	1.4 (2.2)	1.4 (2.0)	ns
Sitting time at work (h/day), mean (SD)	6.4 (2.6)	6.4 (2.6)	ns
Sitting time in cars (h/day), mean (SD)	0.4 (0.8)	0.4 (0.7)	ns
Sitting time in PT (h/day), mean (SD)	0.5 (0.7)	0.4 (0.7)	ns
**Perceived environment attributes, mean (SD)**			
Land use mix diversity	3.0 (0.9)	3.0 (0.9)	ns
Land use mix access	2.9 (0.6)	2.9 (0.6)	ns
Street connectivity	2.8 (0.7)	2.8 (0.7)	ns
Walking and cycling facilities	2.5 (0.7)	2.5 (0.7)	ns
Aesthetics	2.3 (0.7)	2.3 (0.7)	ns
Crime safety	3.0 (0.5)	3.0 (0.5)	ns
**Walk Score®, %**			
Car-dependent (0–69)	–	32.4	
Somewhat walkable (70–89)	–	33.3	
Very walkable (90–100)	–	34.3	

*SD* standard deviation, *ns* non-significant, *PT* public transport.

^a^Difference across subsample categories was tested using x^2^ for categorical variables and t-tests for continuous variables.

**Table 2:** Spearman’s correlations of workplace neighbourhood built-environment attributes.

	a	b	c	d	e	f	g
a. land use mix diversity	1.00						
b. land use mix access	0.52	1.00					
c. street connectivity	0.30	0.49	1.00				
d. walking and cycling facilities	0.22	0.29	0.29	1.00			
e. aesthetics	0.20	0.15	0.15	0.44	1.00		
f. crime safety	0.26	0.46	0.30	0.23	0.07	1.00	
g. Walk Score®	0.38	0.50	0.32	0.22	0.12	0.27	1.00

All the correlations were significant (p < 0.001).

The sample size for the correlations between perceived workplace neighbourhood attributes was 2137 and that for the correlations between Walk Score® and perceived attributes was 1163.

Table 3 presents the associations of workplace neighbourhood built-environment attributes with sitting time at work, in cars, and in public transport. After adjusting for all covariates, including total physical activity, those who perceived their workplace neighbourhoods higher in land-use diversity and access, higher in street connectivity, and safer from crime tended to spend longer time sitting at work. One SD increment in land use mix diversity, land use mix access, street connectivity, and crime safety was associated with 8.6, 15.2, 10.0, and 14.8 more minutes of daily sitting time at work, respectively. Associations were not significant for perceived walking/cycling facilities and aesthetics and any categories of Walk Score®.

**Table 3:** Associations of sitting time at work, in cars, and in public transport with workplace neighbourhood built-environment attributes.

Workplace neighbourhood built-environment attributes	Sitting at work^a^			Sitting in cars^b^			Sitting in public transport^b^		
	β	(95% CI)	p	β	(95% CI)	p	β	(95% CI)	p
**Perceived measures (z-score; n = 2137)**									
Land use mix diversity	**8.6**	**(2.1, 15.1)**	**0.010**	**− 7.3**	**(− 9.3, − 5.3)**	**< 0.001**	**2.0**	**(0.1, 4.0)**	**0.044**
Land use mix access	**15.2**	**(9.0, 21.4)**	**< 0.001**	**− 11.1**	**(− 13.0, − 9.3)**	**< 0.001**	**5.6**	**(3.7, 7.4)**	**< 0.001**
Street connectivity	**10.0**	**(3.7, 16.2)**	**0.002**	**− 4.9**	**(− 6.8, − 3.0)**	**< 0.001**	1.6	(**− **0.3, 3.5)	0.10
Walking and cycling facilities	5.4	(**− **0.9**, **11.7)	0.095	**− 5.4**	**(− 7.4, − 3.5)**	**< 0.001**	**3.8**	**(1.9, 5.7)**	**< 0.001**
Aesthetics	**− **0.9	(**− **7.2, 5.3)	0.77	**− 2.5**	**(− 4.4, − 0.6)**	**0.010**	1.8	(**− **0.1**, **3.7)	0.064
Crime safety	**14.8**	**(8.6, 21.0)**	**< 0.001**	**− 5.5**	**(− 7.4, − 3.6)**	**< 0.001**	1.1	(**− **0.8**, **2.9)	0.27
**Walk Score® (Category; n = 1163)**									
Car**− **dependent (0–69; n = 377)	**− **4.4	(**− **25.0, 16.2)	0.68	**8.8**	**(3.2, 14.4)**	**0.002**	**− 7.5**	**(− 13.7, − 1.4)**	**0.017**
Somewhat walkable (70–89; n = 387)	Ref			Ref			Ref		
Very walkable (90–100; n = 399)	19.4	(**− **1.0, 39.7)	0.063	**− 13.6**	**(− 19.1, − 8.1)**	**< 0.001**	5.1	(**− **1.0, 11.2)	0.10

*β* unstandardised regression coefficient (minutes/day) corresponding to 1 SD increment in perceived attributes and relative to the workplaces located in somewhat walkable neighbourhoods, *CI* confidence interval.

^a^Models were adjusted for gender, age group, marital status, educational level, individual annual income, physical activity duration, work hours per week, and workplace size.

^b^Models were adjusted for gender, age group, marital status, educational level, individual annual income, physical activity duration, driving licence, work hours per week, and workplace size.

Figures highlighted in bold indicate statistically significant findings (p < 0.05).

Covariate-adjusted models also showed that all the perceived workplace neighbourhood environmental attributes were negatively associated with sitting time in cars. For sitting in public transport, three attributes (land use mix diversity, land use mix access, and walking/cycling facilities) were positively associated. The strongest association was found in land use mix access for both sitting in cars (β = − 11.1; 95% CI − 13.0, − 9.3) and sitting in public transport (β = 5.6; 95% CI 3.7, 7.4). Walk Score® was associated with sitting in cars (both car-dependent and very walkable areas showing significant regression coefficients) and in public transport (only car-dependent areas significantly different from somewhat walkable areas).

## Discussion

This study investigated the associations of perceived and objective workplace neighbourhood built-environment attributes with sitting time at work and for transport among desk-based workers in Japan. Contrary to the expectation, perceptions of workplace neighbourhoods to be more supportive of walking were generally associated with the higher sitting time at work. Greater neighbourhood walkability indicated by perceptions of higher land use mix diversity and access, street connectivity, and crime safety was associated with longer sitting at work. The potential explanation underlying the association is that workplaces in high walkable areas such as urban centres with better diversity and access to destinations and connected streets tend to have occupation types involving longer workplace sitting time. Alternatively, this may be attributable to the time saved from travelling from the workplace to and from different destinations with connected streets in the workplace neighbourhood during work breaks. Workers may spend some time outside their workplace (e.g., for errands and lunch breaks). It is possible that such breaks can be shorter in more walkable workplace neighbourhoods, which can lead to longer sitting at work. A previous study from Japan also found that living in more walkable residential neighbourhoods was associated with longer indoor sitting time, potentially due to reduced time spent in commuting and errands^21^. Another recent study conducted in Japan found that Walk Score® was positively associated with objectively measured sedentary behaviours^22^. Our findings may be particular to the Asian work environment and workplace culture. Further research needs to check whether the same relationships are observed in non-Asian contexts, where workplace culture may be different.

There were no statistically significant associations of Walk Score® with sedentary time at work after adjusting for covariates. Walk Score® was moderately correlated with land use mix access (rho = 0.50) and with land use mix diversity (rho = 0.38). Although a positive association was found for perceived access to diverse destinations around the workplace and sitting at work, the association for a similar objective measure was not significant. These findings suggest a discrepancy between perceived access to destinations and objectively measured availability of destinations. It is possible that participants may have limited knowledge of their workplace neighbourhoods (e.g., between the workplace and nearby public transit stops), while Walk Score® is derived using all destinations surrounding the workplace. Such mismatch between perception and reality is known to exist for measures related to land use^23^. However, if our interpretation for perceived urban design measures is correct (i.e., workplaces where workers spend longer time sitting tend to be located at walkable neighbourhoods), we could expect similarly positive associations for Walk Score®. Future studies need to explore this inconsistency to understand mechanisms through which built-environment attributes are related to workplace sitting time.

This study found that more walkable workplace neighbourhoods were associated with less sitting time in cars, consistent with previous study findings^24,25^. We also found such workplace neighbourhoods to be conducive to longer sitting time in public transport. These findings suggest that activity-supportive workplace neighbourhoods would discourage longer car use and promote public transport use, implying the use of combining active modes of travel such as walking and cycling. Integrating the findings of sitting at work and sitting for transport, we could argue that those who work in activity-supportive workplace neighbourhoods may sit longer at work. However, their longer workplace sitting may be mitigated by a shorter amount of car use. Given that Japanese workers spent on average about 1 h and 20 min commuting per day^26^, non-car commuting is likely to involve a considerable amount of standing and walking, which may be a reason for longer sitting at work among those who work in walkable neighbourhoods. Future research needs to examine whether and to what extent sitting behaviours while commuting and at work may compensate each other.

This study has some limitations. First, the non-representative sample of participants, due to the nature of the internet recruitment strategy and self-selection of participants^27,28^, may limit the generalisability. However, participants were randomly selected to be equally distributed across gender- and age groups; these approaches may reduce the selection bias due to the demographic characteristics to some extent. In addition, it was found that participants worked in diverse workplaces in terms of size and location (from car-dependent to very walkable areas). This suggests that there was a certain level of variation in the workplaces and their neighbourhoods, even though an internet survey was used for data collection. Second, the ANEWS environmental-perceptions instrument was specifically developed for assessing urban design attributes of residential neighbourhoods rather than workplace neighbourhoods, and some attributes may not apply to be used in the workplace-neighbourhood context. However, we found that the subscales had acceptable test–retest reliability and internal consistency (except for the traffic safety subscale, which was excluded). Third, the self-reported measures of sitting time and covariates may be subject to recall bias. Additionally, sitting for transport takes place not only within but also beyond workplace neighbourhoods. Unlike sitting at work, there was no strict correspondence between where behaviours took place and where environmental attributes were measured. However, strong and consistent associations of sitting time in cars with workplace neighbourhood environmental attributes were observed (both for perceived attributes and Walk Score®). This suggests that urban design attributes around the workplace may be strong determinants of car use. Fourth, Walk Score® of participants’ workplace was not available to 45% of the sample, mainly due to participants being unable to recall the 7-digit postcode of their workplace. Although participants without Walk Score® did not differ from those with Walk Score® in terms of their mean sitting time and environmental perceptions, some differences in the demographic characteristics and the size of workplace between the two groups; the results for Walk Score® may be biased if missing of Walk Score® occurred in a non-random manner. Fifth, this study may be limited by a lack of comprehensive work-related information, such as job type (management, professional, clerical), workplace policies (e.g., encouraging standing breaks), and workplace indoor settings (e.g., availability of height-adjustable workstations), which may confound the relationships examined. Some participants may have worked from home. However, this is expected to have a minimal impact on the findings, given that only 7.4% of workers worked from home in 2019 in Japan^29^. Research has shown that both the volume and patterns of sitting are related to health^30^. Further studies should consider measuring bouts of sitting behaviour to better understand whether workplace neighbourhood environments are associated with sitting patterns. Finally, as this was a cross-sectional study, causal associations cannot be inferred. Longitudinal studies, such as workplace relocation studies, are required to examine the causal associations.

## Conclusions

Desk-based workers who perceived aspects of their workplace neighbourhood as more walkable reported higher amounts of time spent sitting at work and in public transport but lower amounts of sitting time in cars on workdays. Different strategies may need to be implemented to reduce workers’ sedentary time. For those working in neighbourhoods supportive of walking (e.g., urban centres), workers may be encouraged to take advantage of opportunities outside of the workplace to reduce and interrupt workplace sitting time. For those working in less walkable neighbourhoods, workplace interventions to support active modes of transport (e.g., facilities for cyclists, incentives for public transport use) may help reduce sitting in cars.

## Supplementary Information

Supplementary Information.

## Data Availability

Anonymised data are available from the corresponding author on reasonable requests.
