# Supplementary material for: Workplace neighbourhood built-environment attributes and sitting at work and for transport among Japanese desk-based workers
Source: Sci Rep. 2022 Jan 7;12:195. doi: 10.1038/s41598-021-03071-8 (PMC8741887; doi:10.1038/s41598-021-03071-8)
Supplement: Supplementary file 1 — Supplementary Information. [file 41598_2021_3071_MOESM1_ESM.docx]

# Workplace Neighbourhood Built-Environment Attributes and Sitting at Work and for Transport among Japanese Desk-Based Workers

Chien-Yu Lin^1*^, Mohammad Javad Koohsari^2,3^, Yung Liao^2,4^, Kaori Ishii^2^, Ai Shibata^5^, Tomoki Nakaya^6^, Gavin R. McCormack^2,7^, Nyssa Hadgraft^8^, Takemi Sugiyama^8,9^, Neville Owen^8,9^, Koichiro Oka^2^

^1^ Graduate School of Sport Sciences, Waseda University, Tokorozawa, Japan

^2^ Faculty of Sport Sciences, Waseda University, Tokorozawa, Japan

^3^ Melbourne School of Population and Global Health, The University of Melbourne, Melbourne, Australia

^4^ Department of Health Promotion and Health Education, National Taiwan Normal University, Taipei, Taiwan

^5^ Faculty of Health and Sport Sciences, University of Tsukuba, Tsukuba, Japan

^6^ Graduate School of Environmental Studies, Tohoku University, Sendai, Japan

^7^ Department of Community Health Sciences, Cumming School of Medicine, University of Calgary, Calgary, Canada

^8^ Centre for Urban Transitions, Swinburne University of Technology, Melbourne, Australia

^9^ Behavioural Epidemiology Laboratory, Baker Heart & Diabetes Institute, Melbourne, Australia

***Correspondence:**

Chien-Yu Lin, PhD Candidate

Graduate School of Sport Sciences, Waseda University, Tokorozawa, Japan

2-579-15 Mikajima, Tokorozawa, Saitama, Japan 359-1192

Email: [chienyulin@akane.waseda.jp](mailto:chienyulin@akane.waseda.jp)

Telephone: +81-4-2947-7189

# Supplementary Table 1.The English-translated items of the Japanese sedentary behaviour questionnaire

In the last week (seven days), on how many hours and minutes do you do the domain-specific sedentary behaviour listed below on workdays and non-workdays, respectively? Please select all applicable behaviours and provide the total time of the day for each. If the amount of time you spend sitting varies from day to day, please provide the average time per day.

|  | Workdays | Non-workdays |
| --- | --- | --- |
| Being transported to and from a place by car | Total time of the day  _____hours _____minutes | Total time of the day  _____hours _____minutes |
| Using public transport | Total time of the day  _____hours _____minutes | Total time of the day  _____hours _____minutes |
| At work | Total time of the day  _____hours _____minutes | Total time of the day  _____hours _____minutes |
| Watching television, videos, and DVDs | Total time of the day  _____hours _____minutes | Total time of the day  _____hours _____minutes |
| Using a computer, cell phone, or tablet PC outside of working hours | Total time of the day  _____hours _____minutes | Total time of the day  _____hours _____minutes |
| In leisure time (excluding watching television, videos, and DVDs) | Total time of the day  _____hours _____minutes | Total time of the day  _____hours _____minutes |

# Supplementary Table 2. The English-translated items of the modified Abbreviated Neighborhood Environment Walkability Scale Japanese version for workplace neighbourhood built-environment attributes

1. Land use mix diversity: About how long would it take to get from your workplace to the nearest businesses or facilities listed below if you walked to them? Please select the answer that best applies to you.

|  | 1-5 min | 6-10 min | 11-20 min | 21-30 min | 31+ min | don’t know |
| --- | --- | --- | --- | --- | --- | --- |
| 1. convenience/grocery store |  |  |  |  |  |  |
| 1. supermarket |  |  |  |  |  |  |
| 1. laundry/dry cleaners |  |  |  |  |  |  |
| 1. clothing store |  |  |  |  |  |  |
| 1. post office |  |  |  |  |  |  |
| 1. library |  |  |  |  |  |  |
| 1. book store |  |  |  |  |  |  |
| 1. fast food restaurant (e.g., hamburger, beef bowl, and stand-up-eating noodle stalls) |  |  |  |  |  |  |
| 1. coffee place |  |  |  |  |  |  |
| 1. bank |  |  |  |  |  |  |
| 1. non-fast food restaurant |  |  |  |  |  |  |
| 1. pharmacy/drug store |  |  |  |  |  |  |
| 1. salon/barber shop |  |  |  |  |  |  |
| 1. bus or train stop |  |  |  |  |  |  |
| 1. park |  |  |  |  |  |  |
| 1. gym or fitness facility |  |  |  |  |  |  |

1. Land use mix access: Please select the answer that best applies to your workplace neighbourhood (within a 10- to 15-minute walk from your workplace).

|  | strongly disagree | somewhat disagree | somewhat agree | strongly agree |
| --- | --- | --- | --- | --- |
| 1. Stores are within easy walking distance of my workplace. |  |  |  |  |
| 1. Parking is difficult in the shopping areas around my workplace. |  |  |  |  |
| 1. There are many places (stores, post offices, and public facilities) to go within easy walking distance of my workplace. |  |  |  |  |
| 1. It is easy to walk to a transit stop (bus, train) from my workplace. |  |  |  |  |
| 1. The streets in my workplace neighbourhood are hilly, making my workplace neighbourhood difficult to walk in. (reversely coded) |  |  |  |  |
| 1. There are major freeways, railway lines, or rivers to walking in my workplace neighbourhood that make it hard to get from place to place. (reversely coded) |  |  |  |  |

1. Street connectivity: Please select the answer that best applies to your workplace neighbourhood (within a 10- to 15-minute walk from your workplace).

|  | strongly disagree | somewhat disagree | somewhat agree | strongly agree |
| --- | --- | --- | --- | --- |
| 1. The streets in my workplace neighbourhood do not have many cul-de-sacs. |  |  |  |  |
| 1. The distance between intersections in my workplace neighbourhood is usually short (100 meters or less). |  |  |  |  |
| 1. There are many alternative routes for getting from place to place in my workplace neighbourhood. (I don't have to go the same way every time.) |  |  |  |  |

1. Availability and quality of walking/cycling infrastructures: Please select the answer that best applies to your workplace neighbourhood (within a 10- to 15-minute walk from your workplace).

|  | strongly disagree | somewhat disagree | somewhat agree | strongly agree |
| --- | --- | --- | --- | --- |
| 1. There are sidewalks on most of the streets in my workplace neighbourhood. |  |  |  |  |
| 1. Sidewalks are separated from the road/traffic in my workplace neighbourhood by guardrails and steps. |  |  |  |  |
| 1. Sidewalks are separated from the road/traffic in my workplace neighbourhood by parked cars. |  |  |  |  |
| 1. There is a grass/dirt strip that separates the streets from the sidewalks in my workplace neighbourhood. |  |  |  |  |

1. Aesthetics: Please select the answer that best applies to your workplace neighbourhood (within a 10- to 15-minute walk from your workplace).

|  | strongly disagree | somewhat disagree | somewhat agree | strongly agree |
| --- | --- | --- | --- | --- |
| 1. There are trees along the streets in my workplace neighbourhood. |  |  |  |  |
| 1. There are many interesting things to look at while walking in my workplace neighbourhood. |  |  |  |  |
| 1. There are many attractive natural sights in my workplace neighbourhood. |  |  |  |  |
| 1. There are attractive buildings/homes in my workplace neighbourhood. |  |  |  |  |

1. Crime safety: Please select the answer that best applies to your workplace neighbourhood (within a 10- to 15-minute walk from your workplace).

|  | strongly disagree | somewhat disagree | somewhat agree | strongly agree |
| --- | --- | --- | --- | --- |
| 1. My workplace neighbourhood streets are well lit at night. |  |  |  |  |
| 1. Walkers and bikers on the streets in my workplace neighbourhood can be easily seen by people in their homes. |  |  |  |  |
| 1. There is a high crime rate in my workplace neighbourhood. (reversely coded) |  |  |  |  |
| 1. The crime rate in my workplace neighbourhood makes it unsafe to go on walks during the day. (reversely coded) |  |  |  |  |
| 1. The crime rate in my workplace neighbourhood makes it unsafe to go on walks at night. (reversely coded) |  |  |  |  |

# Supplementary Table 3. Intraclass correlation coefficients (ICCs)^a^ and 95% confidence intervals (CIs) of the six subscales of the modified Abbreviated Neighborhood Environment Walkability Scale Japanese version

| Subscales | ICC | (95% CI) | | p value |
| --- | --- | --- | --- | --- |
| Land use mix diversity | 0.87 | (0.82 , | 0.90) | <0.001 |
| Land use mix access | 0.82 | (0.77 , | 0.87) | <0.001 |
| Street connectivity | 0.57 | (0.43 , | 0.67) | <0.001 |
| Walking and cycling facilities | 0.77 | (0.70 , | 0.83) | <0.001 |
| Aesthetics | 0.73 | (0.65 , | 0.80) | <0.001 |
| Crime safety | 0.78 | (0.71 , | 0.83) | <0.001 |

^a^ The ICCs were interpreted as indicating low (< 0.4), moderate (≥ 0.4 to < 0.75), and high reliability (≥ 0.75).

# Supplementary Table 4. Comparison of the characteristics of participants who reported valid Walk Score^®^ and those who did not report valid Walk Score^®^

| Characteristics | Those who reported valid Walk Score^®^  (n=1,163) | |  | Those who did not report valid Walk Score^®^  (n=974) | | p |
| --- | --- | --- | --- | --- | --- | --- |
|  | N or mean | % or SD |  | N or mean | % or SD |  |
| Gender |  |  |  |  |  | 0.029 |
| Men | 583 | 50.1% |  | 442 | 45.4% |  |
| Women | 580 | 49.9% |  | 532 | 54.6% |  |
| Age group (year) |  |  |  |  |  | 0.001 |
| 20-29 | 243 | 20.9% |  | 261 | 26.8% |  |
| 30-39 | 278 | 23.9% |  | 255 | 26.2% |  |
| 40-49 | 320 | 27.5% |  | 230 | 23.6% |  |
| 50-59 | 322 | 27.7% |  | 228 | 23.4% |  |
| Marital status |  |  |  |  |  | 0.002 |
| Married | 610 | 52.5% |  | 577 | 59.2% |  |
| Not married | 553 | 47.5% |  | 397 | 40.8% |  |
| Educational level |  |  |  |  |  | ns |
| Have tertiary education | 1005 | 86.4% |  | 833 | 85.5% |  |
| Below tertiary education | 158 | 13.6% |  | 141 | 14.5% |  |
| Annual income (yen) |  |  |  |  |  | 0.011 |
| ≥ 4,000,000 yen | 570 | 49.0% |  | 531 | 54.5% |  |
| < 4,000,000 yen | 593 | 51.0% |  | 443 | 45.5% |  |
| Driving licence |  |  |  |  |  | ns |
| Yes | 1049 | 90.2% |  | 867 | 89.0% |  |
| No | 114 | 9.8% |  | 107 | 11.0% |  |
| Work hours per week, mean (SD) | 45.0 | 13.8 |  | 44.4 | 12.8 | ns |
| Workplace size |  |  |  |  |  | <0.001 |
| Small (≤ 29 employees) | 345 | 29.7% |  | 165 | 16.9% |  |
| Medium (30-99 employees) | 160 | 13.8% |  | 150 | 15.4% |  |
| Large (≥ 100 employees) | 626 | 53.8% |  | 623 | 64.0% |  |
| Missing | 32 | 2.8% |  | 36 | 3.7% |  |
| Sitting time at work (hour/day), mean (SD) | 6.4 | 2.6 |  | 6.4 | 2.6 | ns |
| Sitting time in car (hour/day), mean (SD) | 0.4 | 0.7 |  | 0.4 | 0.8 | ns |
| Sitting time in public transport (hour/day), mean (SD) | 0.4 | 0.7 |  | 0.5 | 0.8 | ns |
| Perceived environment attributes, mean (SD) |  |  |  |  |  |  |
| Land use mix diversity | 3.0 | 0.9 |  | 2.9 | 0.9 | ns |
| Land use mix access | 2.9 | 0.6 |  | 2.9 | 0.6 | ns |
| Street connectivity | 2.8 | 0.7 |  | 2.8 | 0.7 | ns |
| Walking and cycling facilities | 2.5 | 0.7 |  | 2.6 | 0.7 | ns |
| Aesthetics | 2.3 | 0.7 |  | 2.3 | 0.7 | ns |
| Crime safety | 3.0 | 0.5 |  | 3.0 | 0.5 | ns |

Note: SD, standard deviation; ns, non-significant

^a^ Difference across subsample categories was tested using x^2^ for categorical variables and t-tests for continuous variables.
